# The weight of words: Applying concreteness to elite media language

**DOI:** 10.1371/journal.pone.0359092

**Published:** 2026-10-01

**Authors:** Donald Snyder

**Affiliations:** Wesleyan University, Middletown, Connecticut, United States of America; Educational Testing Service: ETS, UNITED STATES OF AMERICA

## Abstract

This study introduces lexical “concreteness” – a psycholinguistic measure of vivid language – as a scalable tool for political and news text analysis. Using a dictionary of approximately 40,000 English word lemmas rated for concreteness, this construct is applied to more than two million *New York Times* articles published between 2000 and 2025. Exploratory analyses show that average concreteness declines over time in headlines and abstracts and varies systematically across material categories, document formats, print page placement, and article length. Descriptive formats, including news reports and biographies or obituaries, generally use more concrete language than editorial or opinion content. Taken together, these analyses suggest that concreteness may be a meaningful linguistic feature in the political news context, capturing unique stylistic features in media language.

## Introduction

Political language varies in the way it is expressed, sometimes evoking strong responses. Even within the same political party, rhetorical styles may differ. Republican slogans like “build a wall” or “drain the swamp” invoke vivid, material imagery, while terms like “woke” or “law and order” rely on symbolic abstraction. Likewise, Democratic-associated phrases such as “tax the rich” or “Black Lives Matter” point to identifiable actors and actions, while slogans like “change we can believe in” or “let’s win this” appeal to generalized ideals. These stylistic differences are not just incidental – they can shape the way that political messages are interpreted, remembered, and acted upon [1–3].

These phrases are just one illustration of a broader pattern: variations in the concreteness of words are present in elite rhetoric, media framings, and the ways that members of the mass public describe political issues, and may have important implications for political behaviors. Certain language might tend towards being more symbolic and abstract, while other language is more tangible and concrete. Although political scientists routinely study text through lenses of issue content [4], partisanship [5], and sentiment [6], they rarely quantitatively measure meaningful stylistic differences within the words themselves. This paper applies a scalable, dictionary-based measure of linguistic concreteness to elite media coverage in the *New York Times*, analyzing news features associated with higher and lower levels of the construct.

Concreteness of words is a linguistic feature that measures psychologically tangible qualities within verbal or textual information [7]. This construct is described as an important variable in a variety of different arenas. Extant psychological research suggests important cognitive implications for concrete imagery and text comprehension [8]. Evidence suggests that concrete imagery tends to increase recall of objects in memory [9,10]. Other research describes how, as the psychological distance between two groups decreases, individual-level cognition becomes more concrete [11]. Temporal distance and valence are also shown to have important relationships with linguistic concreteness [12,13]. Literature on goal-oriented behavior suggests that those who tend to view goals and actions concretely perceive more dissimilarity between themselves and others, and feel less empathy for them [14]. These veins of research tend to view concreteness as a trait for priming [11] or as something to be hand-coded in small-n datasets [15], as opposed to the automated, large-scale approach presented in this paper.

There are indications that the concreteness construct may have distinct relevance to political-scientific research. Varying levels of concreteness or abstraction have demonstrable impacts on how seriously political information is taken. Duan et al. [16] find evidence that showing respondents images about the concrete (rather than abstract) impact of climate change tends to increase the intention to mitigate climate change and increase levels of concern among conservatives, moderates, and liberals alike. Yogeeswaran and Dasgupta [17] find evidence that priming concrete information about Hispanic Americans increased prejudicial attitudes towards them relative to a control. Research suggests that priming concrete information may lead to perceptions of increased differences between dyads of political groups and countries [18]. Other literature suggests that political actors may support abstract principles of equality between races, despite opposing tangible or concrete policies to achieve goals of equality [19–24].

The analyses in this paper attempt to unpack the unique dynamics of the concreteness construct in the political media context. The concreteness construct could potentially offer new leverage on questions such as why certain political messages are more memorable [25], why some appeals mobilize low-engagement voters [26], and how styles of language moderate policy frames [27]. Concreteness may also open new paths for understanding stylistic variations in elite rhetoric – such as populist versus technocratic speech [28] or personalized campaign strategies [1,29]. Unlike tools that capture *what* is being said, a concreteness-based lexical approach may help reveal *how* political messages are conceptualized and conveyed – and could serve as an operationalization of vividness, specificity, and experiential qualities that shape how individuals interpret, remember, and personalize political content.

## Concreteness in text data

Concreteness of words can be operationalized using several different types of methodologies. Semin and Fiedler [30] provide a way to operationalize concreteness in text using their “Linguistic Categorization Model.” The Linguistic Categorization Model utilizes categories for parts of speech, including (in order of increasing abstractness) the presence of descriptive action verbs, interpretive action verbs, state verbs, and adjectives. This is largely applied as a hand-coded methodology [15]. On the other hand, automatic models build on the work of Brysbaert et al. [7], who developed a vast database of words, rated on a spectrum from abstract to concrete. This method of automatic categorization has been used to examine social media outcomes [31], argument strategies [32], and contextual influences such as temporal distance [12] and valence [13]. However, due to methodological or topical constraints, most of these papers either limit the range of words used from the dictionary or apply the measure to narrowly defined events or contexts.

This paper uses the database of Brysbaert et al. [7] to operationalize the concreteness construct. This database is methodologically rigorous, stemming from millions of ratings of nearly 40,000 words from online respondents (scale from 1–5, 1 = most abstract, 5 = most concrete), based on visual and haptic experiences they associate with these words [7]. Unigrams and bigrams that tend to be rated as concrete describe specific concepts with clear visual imagery (“baseball bat” – 5, “gunfire” – 4.66, “ballot” – 4.73), while those that are abstract are more nebulous and interpretive (“spirituality” – 1.14, “ideology” – 1.62, “power” – 2.04).

While the Brysbaert et al. [7] dictionary is well cited in psycholinguistic research, it remains underutilized in political contexts. This is likely due to disciplinary gaps and lack of construct clarity within political communication. This paper helps to clarify this concreteness construct in political media text, using a large corpus of over two million *New York Times* articles. The concreteness measure is further examined through article metadata comparisons, which help to characterize the nature of the construct in the media context. These exploratory analyses weigh the value of concreteness as a politically relevant linguistic feature and help to better characterize its potential future applications within this domain.

## Methods

### Computing concreteness

The primary dataset used in this study is the publicly available *New York Times* article database compiled by Singh [33], (There were some omissions in this database, which were addressed by re-scraping articles using the *New York Times* API.) which includes over two million articles from 2000 to the present. Metadata fields include the article’s headline, abstract, snippet, and lead paragraph text, as well as date, section, print page, and other contextual attributes (The “abstract” field refers to a short article-summary field supplied in the New York Times metadata, rather than a scholarly abstract). To compute concreteness, article text is scored using the Brysbaert et al. [7] dictionary of nearly 40,000 word lemmas, each rated on a 1–5 scale (1 = most abstract, 5 = most concrete). Text was parsed to calculate an average concreteness score based on unigram and bigram matches against the dictionary (example shown in Fig 1).

**Fig 1:**
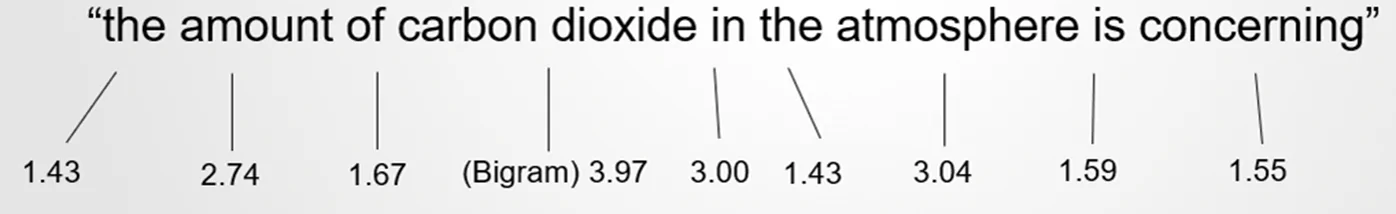
Concreteness example rating.

Before dictionary matching, all text was converted to lowercase and punctuation was removed, while contractions were retained because some contracted forms of words are included in the dictionary [7]. Tokens were evaluated in their original order so that both unigram and bigram matches could be identified. When a bigram appeared in the dictionary, the bigram score was prioritized and its constituent words were skipped as unigrams, preventing double counting. Tokens not found in the dictionary were excluded from both the summed score and its denominator, to avoid influencing the mean response rating. Given the breadth of the dictionary, unmatched tokens consisted primarily of proper names, acronyms, and misspelled words, and pre-processing reduced mismatches caused by capitalization and punctuation. Because the dictionary includes whole and inflected word forms, neither stemming nor lemmatization was applied to the concreteness score used in primary analyses. Word coverage across the full *New York Times* database is fairly strong, with 73.8% of words having a direct match to the database (69% for headlines, 75% for abstract, 77% for lead paragraph). As an additional robustness test, the same analyses in the results section were also performed using a score that rated only content words and, separately, a score that did not rate stop words. In each case, none of the directions of the relationships changed, so the results going forward are reported from the ratings using the full corpus of words.

### Meta-Informational Factors of Concreteness

The concreteness algorithm is applied across four *New York Times* article text fields: headline, abstract, snippet, and lead paragraph. Snippet text was excluded from later analyses due to its redundancy with abstract text (see Fig 6 in S1 Appendix). Text entries were cleaned to remove blank fields, outliers, and other processing errors. The resulting dataset provides average concreteness scores for each article text type, enabling descriptive comparisons across a variety of metadata attributes for the measure of concreteness.

To examine how this measure relates to contextual features, concreteness variations are analyzed in relation to article metadata. These fields include:

- **Temporal variables**: Year, month, and publication date
- **Positional features**: Page of the paper that the article is printed on and full-article’s word count
- **Content domains**: News desk, section name, and material type
- **Document format**: Document type (e.g., article, audio, multimedia)

Within the “content domains” category, news desk and section name fields described the writing team or section of the paper where the article was posted. News desk and section name largely overlapped, and were recoded into topical domains including arts and culture/entertainment (e.g., film, books, fashion), business/financial, editorial/opinion, health, national/regional, science/technology, sports, and travel. The material type field, which describes the editorial purpose or structure of the content, was recoded into categories such as analysis (in-depth reporting or thematic discussion), biography/obituary, correction/editor’s note, editorial/opinion, interview, list (e.g., “Top 10” or summary items), news/report, and review articles (e.g., books, movies). Lastly, document type refers to the article’s media format – whether it is a standard article, audio piece, or multimedia entry.

No a priori hypotheses were formulated for these analyses, though a host of relationships between metadata and the concreteness scores are systematically examined. This approach reflects the exploratory aim of the paper: to better characterize the descriptive contours of the concreteness construct. As a first step, metadata features serve as a testing ground for evaluating what types of language this construct is sensitive to in a political media context. The analyses assess how average concreteness varies across time, topic, placement in the paper, article length, and medium, drawing on the full population of *New York Times* articles published from 2000 onward. Because these analyses are performed on a full population of metadata, they are reported as mean differences, slopes per year, etc., rather than using inferential statistics.

The *New York Times* is a substantively important case study for understanding professional news language: it is one of the largest and most prominent news organizations in the United States, with a national and international readership and more than 12 million subscribers [34]. Although these features strengthen the usefulness of the case study, the *New York Times* is not necessarily representative of every newspaper or media outlet. However, because of its central role in the news space and the extensive article metadata present through its API, it serves as an important descriptive case, and as such, certain variations observed in concreteness may generalize to broader news or political media contexts.

## Results

Initial trends in *New York Times* text data indicate normal distributions of each article text field, with some differences. Headlines, the shortest average text snippets, tend to display more variance compared to abstract or lead paragraph text (Fig 4 in S1 Appendix). This variance may make headlines a more informative unit of analysis, especially considering that headlines receive demonstrably more attention from article consumers [35].

Trends over time (Fig 2) (The large drop in abstract concreteness around 2005 is presumed to be from digital media transition policies implemented by *New York Times* leadership. This theory is supported somewhat in the exploratory analyses, as all news desk categories show similar drops in concreteness during this period, though more evidence would be needed to fully understand this phenomenon.) show a general decline in average concreteness across abstract (−0.35 points over time), lead paragraph (−0.02 points over time), and headline text (−0.13 points over time). These patterns persist even when controlling for word count. Meanwhile, the prevalence of articles classified as “other” increases over time (Fig 5 in S1 Appendix), potentially indicating a temporal shift towards less topically constrained and more multi-faceted news content.

**Fig 2:**
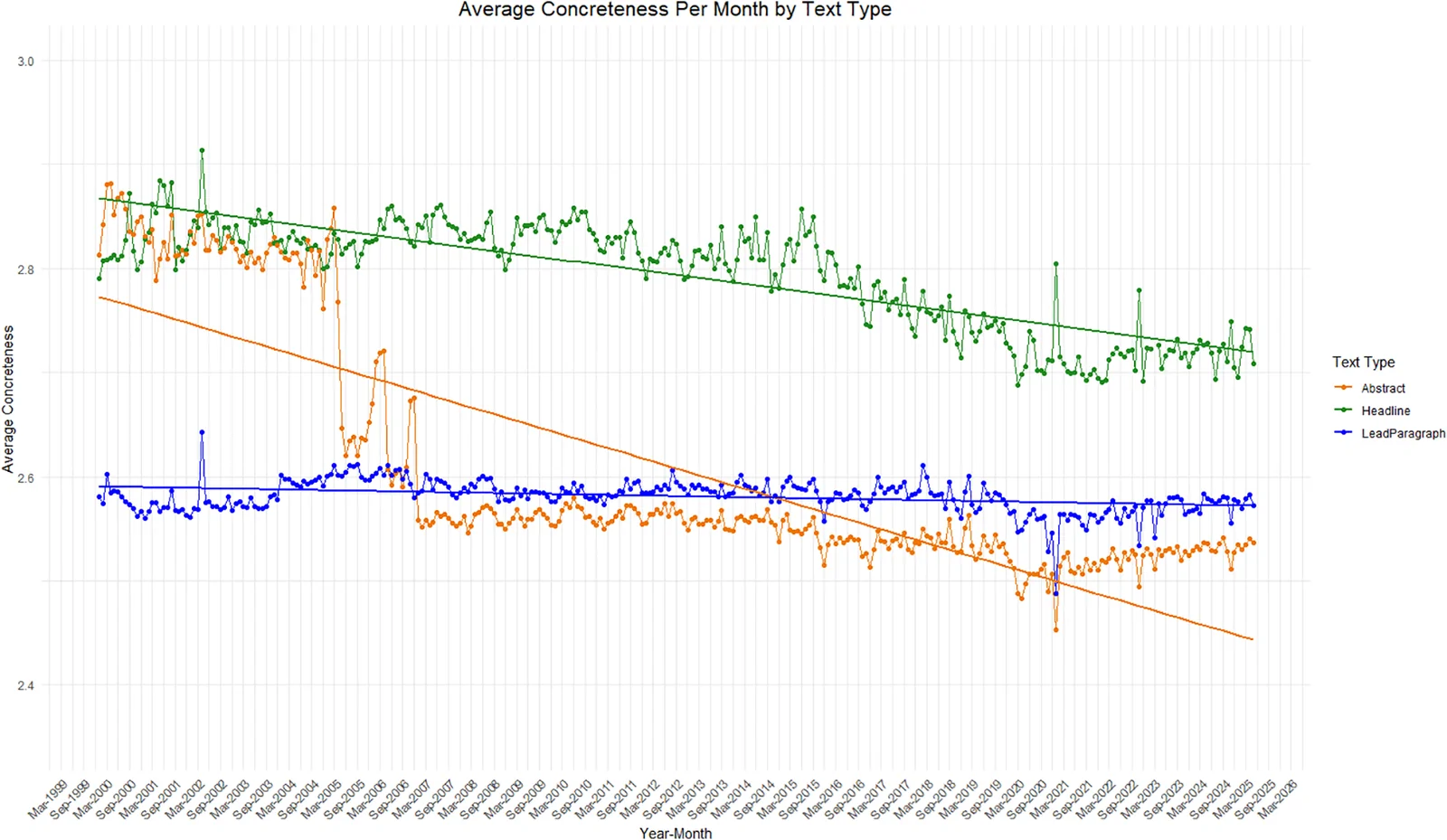
Concreteness over time by text type.

Despite this, category-specific trends indicate that the aggregate decline in concreteness is not solely attributable to changes in the composition of New York Times content. Average headline concreteness also declines within several major categories over the study period. This includes major categories such as the News/Report and Editorial/Opinion entries (Fig 3). The patterns of these categories suggest that both descriptive and opinionated styles of news content experience generally similar temporal trends across time.

**Fig 3:**
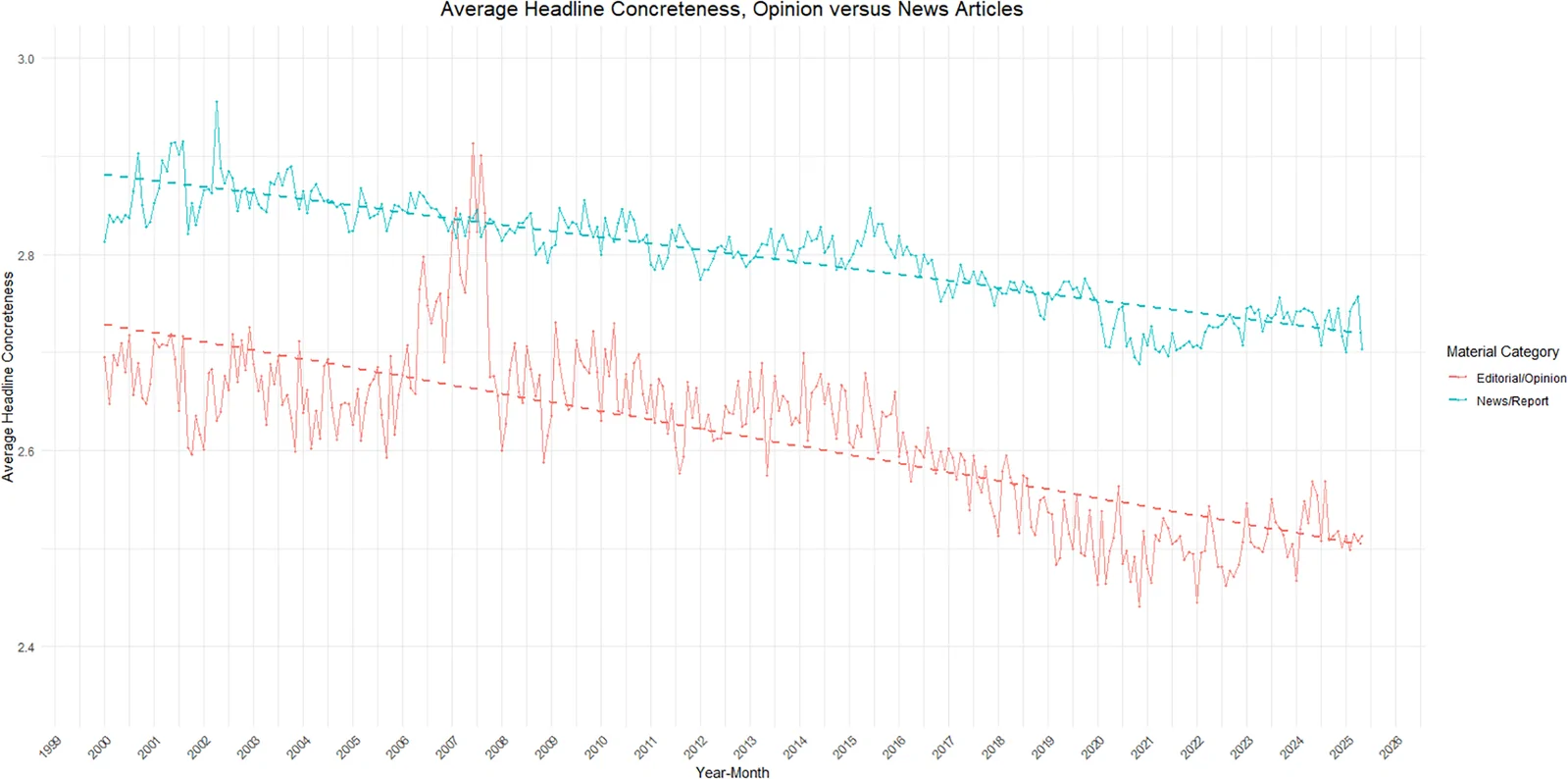
Concreteness over time, opinion versus news articles.

Additional variation appears across the broader material categories (Table 1). Biographies and obituaries are relatively concrete compared to the other categories, which may reflect their emphasis on identifiable people, locations, events, and details from an individual’s life. Analysis entries, by contrast, generally use less concrete language, particularly in their abstracts and lead paragraphs. The pooled averages in Table 1 show that Lists have the highest headline concreteness score at 3.217, while Biography/Obituary entries also rate comparatively highly at 2.892. Editorial/Opinion has the lowest average headline score at 2.625, while Analysis entries have especially low average concreteness in abstracts and lead paragraphs, at 2.490 and 2.460 respectively. These differences suggest that descriptive genres may have different trends in word choice compared to more interpretive work. In this sense, the material-category results complement the news-desk comparisons by indicating that concreteness may vary with both the topic of an entry and the editorial form through which that topic is presented.

**Table 1:** Average concreteness scores by material category.

Material Category	Headline	Abstract	Lead Paragraph
List	3.2165	2.8025	3.0870
Other	2.9771	2.6354	2.6257
Correction/Editor’s Note	2.8607	2.7881	2.6509
Biography/Obituary	2.8918	2.6552	2.6503
News/Report	2.8239	2.6253	2.5975
Analysis	2.7459	2.4899	2.4599
Interview	2.6964	2.7851	2.6439
Review	2.6810	2.7199	2.5961
Editorial/Opinion	2.6250	2.5844	2.4582

While material category captures an entry’s editorial purpose and structure, news desk captures differences across topic domains. Fig 7 in S1 Appendix shows the pooled average headline concreteness scores across *New York Times* news-desk categories. Travel and National/Regional content have the highest average headline scores, at 2.881 and 2.872 respectively, followed by Business/Financial content at 2.840. Editorial/Opinion is notably lower than every other news-desk category, with an average headline score of 2.607 (see Table 3 in S1 Appendix). This pattern is consistent with the idea that concreteness may distinguish language centered on identifiable locations, organizations, transactions, and events from language that evaluates or interprets. These differences suggest that topical and editorial concerns may be associated with differences in linguistic concreteness.

Document format shows a related but more limited pattern. Multimedia entries have the highest average headline concreteness, at 2.992, compared to 2.811 for standard articles and 2.753 for audio entries (Table 2 in S1 Appendix). This difference may reflect a greater need for multimedia headlines to identify specific actors, objects, locations, or events while labeling visual, audio, or interactive content. The difference is present primarily in headlines, as abstract and lead-paragraph differences are relatively limited across document types (For “audio” and “multimedia,” averages for abstract and lead paragraph are identical because they are the same descriptor for these document styles).

For relationships related to paper positioning, headline text is consistently more concrete than either abstract or lead paragraph text. The page of the paper an article appears on has variations in headline (−0.005 points per 10 pages later in the paper), abstract (−0.005 points per 10 pages), and lead paragraph concreteness (0.001 points per 10 pages), with headline and abstract concreteness decreasing as articles appear later in the print edition. Using the full-article word count supplied in the *New York Times* metadata, (Though the full-article word count is present in metadata from the *New York Times* API, the full article text itself is not.) there are conflicting results: headline (−0.083 points per 1,000 words) and abstract text (−0.043 points per 1,000 words) are associated with decreased concreteness as full article word count increases, while the lead paragraph (0.005 points per 1,000 words) has increased concreteness as the article word count increases. Together, these results suggest that longer articles and articles appearing later in the print edition may be more interpretive in their headlines and summaries, though their introductory paragraphs are more grounded and concrete. Each of these broad patterns persists when adjusting for the word count of the metadata fields themselves.

## Discussion

These results suggest that linguistic concreteness may be a useful marker of rhetorical style in political communication. Across analyses, several insights emerge. Concreteness varies across text fields, time periods, news desks, material categories, document formats, print placement, and article length. These patterns indicate that the measure could be sensitive to multiple stylistic and contextual dimensions of news language, though more research is needed to fully understand the causes of these variations.

Across the board, editorial/opinion articles tend to contain text that rates lower in concreteness. Additionally, concreteness has generally decreased across time, in all manner of article text. This pattern may coincide with broader changes in the media landscape, such as a shift toward more abstract or interpretive article framing. Articles tend to rate lower in concreteness as they are placed on later pages within the *New York Times* newspaper. This may be because later excerpts of the paper tend to be where “fluff” pieces are located – and these are likely to be less constrained by rules or norms. This potential explanation may also align with work that describes a considerable portion of modern news content as designed to fill space and be less weighty for the reader [36]. As such, concreteness may have potential implications for measuring varying styles of news content, such as hard, descriptive versus soft, infotainment news [37].

This temporal pattern of decreasing concreteness over time may be situated within broader accounts of marketization, tabloidization, and the digital transformation of news. Evidence suggests that news organizations respond to competitive pressures by changing the structure of the information they present to their audiences [38,39]. Research on hard and soft news similarly describes a long-term movement toward lighter, more entertainment-oriented, and immediately accessible forms of political coverage [37,40]. These pressures may be intensified in digital environments, where audience metrics, limited attention, and competition for visibility encourage outlets to package information in cognitively accessible and attention-grabbing ways [41–43]. Social-media platforms could further reinforce these incentives by algorithmically rewarding content that attracts clicks, shares, and comments [44–46]. The decline in concreteness observed here may be consistent with these broader developments, though the present analyses cannot fully determine whether these trends reflect changing editorial practices, platform incentives, shifts in the composition of coverage, or other changes in the underlying metadata.

These metadata results suggest that concreteness varies along several separate dimensions of New York Times content. Variables like news desk serve as a proxy for measuring differences associated with topical domain. Material type may capture editorial purpose and structure, and document type could measure differences in publication medium. The recurring contrast between more descriptive formats and more interpretive formats suggests that concreteness may be particularly useful for characterizing how journalistic content is presented, even when the substantive subject matter is similar. At the same time, these analyses are exploratory and do not establish that concrete language is necessarily more or less desirable than abstract language – rather, they address broader developments in the ways that news organizations communicate topics.

## Conclusions and future directions

These analyses suggest that a concreteness-based measure may be attuned to key stylistic features of language that are often difficult to quantify. Across *New York Times* text fields and metadata categories, the results show that concreteness captures meaningful differences in how news content is described by media institutions. Though prior work has examined trends in concreteness within psychological domains or around particular events [12,13,31,32], this study extends that research by applying the measure across multiple *New York Times* text fields and metadata categories.

There are some major limitations to the current analyses. First, the *New York Times* may not be broadly representative of trends in media. Trends in concreteness might vary greatly across types of news media, depending on outlet reputation, political leaning, and journalistic style. Additionally, these analyses are exploratory and do not establish causality of associations. Indeed, some patterns of concreteness are somewhat unexplained even in the current analyses, such as the sharp drop in average article abstract concreteness around the year 2005. Analyses of other news sources and alternative methodologies could better illuminate the concreteness construct in the broader media environment, and could provide pre-registered, confirmatory research that would supplement the descriptive analyses of this paper.

These findings open several other paths for future research: assessing rhetorical styles, tracing how contested political concepts evolve in public discourse, and examining how linguistic characteristics influence partisanship, polarization, and understandings of political identities. For example, concreteness could help measure how elite institutions, such as courts, parties, or media outlets, encode political concepts that are later interpreted and redistributed within the mass public. Concreteness might complement popular measures such as sentiment analysis by distinguishing emotionally charged but vague statements from specific, personalized feelings. Concreteness offers a precise yet flexible construct that can supplement and enhance existing models using political text data.

This project illustrates how the psycholinguistic measure of concreteness can be applied to political text, offering conceptual clarity and suggesting unique applications within the political domain. By incorporating this concreteness measure into text-as-data analyses, political scientists may be better able to measure shifts in interpretive elite rhetoric and individuals’ personal resonance with political topics. Broadly, concreteness offers a methodological bridge between psycholinguistics and political science, which may allow scholars to quantify not just *what* political actors say, but *how* their language is grounded in imagery, specificity, and personal meaning.

## Supporting information

S1 Appendix Figs 4 to 7 and Tables 2 & 3. (DOCX)
