# Supplementary material for: The weight of words: Applying concreteness to elite media language
Source: PLoS One. 2026 Oct 1;21(10):e0359092. doi: 10.1371/journal.pone.0359092 (PMC13630233; doi:10.1371/journal.pone.0359092)
Supplement: Supplementary file 1 [file pone.0359092.s001.docx]

Online Appendix For: The Weight of Words: Applying Concreteness to Elite Media Language

Fig 4. Distribution of Concreteness Scores By Text Type

Fig 5. Article News Desk Prevalence Over Time

Fig 6. New York Times Concreteness Correlations

Fig 7. Concreteness By News Desk

| **Document Category** | **Headline** | **Abstract** | **Lead Paragraph** |
| --- | --- | --- | --- |
| Article | 2.8111 | 2.6311 | 2.5900 |
| Audio | 2.7533 | 2.5939 | 2.5939 |
| Multimedia | 2.9922 | 2.6098 | 2.6098 |

Table 2. Average Concreteness Scores by Document Category

| **News Desk Category** | **Headline** | **Abstract** | **Lead Paragraph** |
| --- | --- | --- | --- |
| Travel | 2.8812 | 2.7040 | 2.5934 |
| National/Regional | 2.8718 | 2.6306 | 2.6193 |
| Business/Financial | 2.8397 | 2.5927 | 2.5591 |
| Other | 2.8167 | 2.6035 | 2.5866 |
| Science/Technology | 2.7969 | 2.5609 | 2.5673 |
| Arts & Culture/Entertainment | 2.7686 | 2.6769 | 2.5988 |
| Health | 2.7701 | 2.6933 | 2.5795 |
| Sports | 2.7145 | 2.7099 | 2.5971 |
| Editorial/Opinion | 2.6071 | 2.5045 | 2.4752 |

Table 3. Average Concreteness Scores by News Desk Category
