# Supplementary material for: The weight of words: Applying concreteness to elite media language
Source: PLoS One. 2026 Oct 1;21(10):e0359092. doi: 10.1371/journal.pone.0359092 (PMC13630233; doi:10.1371/journal.pone.0359092)
Supplement: Supplementary file 2 [file pone.0359092.s002.docx]

Response to Reviewers, Editor

I thank the editor and the reviewers for their thoughtful and constructive feedback, and for their time and care when reviewing this manuscript. I appreciate the positive remarks regarding the paper’s potential contribution, and address each of the reviewers/editor’s separate comments in the responses below.

**Reviewer #1**

**Comment 1:** This is a very interesting article, of relevance to public opinion research, as well as to political science, discourse and communication. The data and analysis concerning NYT articles seems to me to be very clear.

**Response 1:** I appreciate the reviewer’s positive assessment of this paper’s contribution.

**Comment 2:** I do not understand what you mean by "abstract" (is this an American term?). What is the "abstract" of a news or opinion article? This can easily be explained.

**Response 2:** I agree that the language regarding the “abstract” field could have been clearer. The updated article has a footnote explaining that: “*The “abstract” field refers to a short article-summary field supplied in the New York Times metadata, rather than a scholarly abstract.”*

**Comment 3:** I have a few bigger concerns:
1. The NYT study is very interesting. But this goes from 1999 to 2026. Can you say more about the sudden change that seems to occur around 2005? Why don't you show the data for news and opinion articles separately? Surely the results must be very different.

**Response 3:** I appreciate the thoughtful comment. In response, I created an additional figure comparing opinion and news articles, showing that they each demonstrate drops in average concreteness over time. With regards to the 2005 sudden change, I performed additional analyses to narrow in on the reason for this phenomenon, such as examining trends by new desk. All news categories showed this drop in abstract concreteness, so I added this to the footnote text: *“The large drop in abstract concreteness around 2005 is presumed to be from digital media transition policies implemented by New York Times leadership. This theory is supported somewhat in the exploratory analyses, as all news desk categories show similar drops in concreteness during this period, though more evidence would be needed to fully understand this phenomenon.”*

**Comment 4:** 2. Since the data from NYT cover 27 years, why is it that the opinion poll data only deal with 2024 and 2025? I do not understand the study design here, and it is not properly explained. This "opinion" study seems quite separate from the other one - unless, of course, you are simply testing the construct, but I think that is not the case… Personally, I feel that you could expand the discussion of the NYT data and leave the opinion poll data for another paper.

**Response 4:** I agree that the opinion poll design portion of this paper is underdeveloped. Because of this, I have dropped the discussion of opinion poll data and added more elaboration about the findings from the New York Times data. This included devoting a paragraph to each of the separate metadata fields, moving analyses from the appendix into the main prose, and performing additional analyses across categories of metadata fields.

**Comment 5:** 3. Your discussion talks about the drop in concreteness in relation to "temporal changes in the media landscape". There is a vast literature on this (trivialisation, tabloidisation, effect of social media) that you need to address.

**Response 5:** I agree that this literature is notably lacking in the previous form of the paper. I have now added a new paragraph in the Discussion section, giving a review of some of this literature, beginning with the sentence *“This temporal pattern of decreasing concreteness over time may be situated within broader accounts of marketization, tabloidization, and the digital transformation of news.”*

**Reviewer #2**

**Comment 1:** The manuscript introduces a valuable application of psycholinguistic concreteness to political text analysis. The core research question is whether concreteness captures meaningful stylistic differences in elite and mass-level political
language, wich is potentially significant for the field. The data sources (New York Times corpus, YouGov surveys) are well-established. The manuscript has strong potential to establish concreteness as a standard tool in political text analysis. The core contribution is valid and valuable, and all remaining issues should be correctable through re-analysis, additional documentation, and tempered conclusions. Methodological and statistical issues must be addressed before publication to meet the standards for rigor, transparency, and reproducibility, and below are specific required revisions.

**Response 1:** I appreciate the reviewer’s positive response to this project, and have made a concerted effort to address each of the methodological, statistical, and documentation issues indicated in the reviewer comments.

**Comment 2:** The New York Times corpus (~2 million articles from 2000–2025) represents a population, not a sample, for claims specifically about NYT articles. Inferential statistics (p-values, t-tests) are conceptually inappropriate for population data because there is no sampling uncertainty to estimate. Remove t-tests and p-values for all NYT population-level analyses (Section 4.1). Replace with descriptive summaries: mean differences, slopes per year, and confidence intervals for measurement precision (not hypothesis testing)

**Response 2:** I appreciate the reviewer’s careful attention to this important detail. I have changed all of the t-tests and p-values and replaced them with descriptive summaries. I also now also clarify the reason for reporting statistics in this manner, stating that: *“Because these analyses are performed on a full population of metadata, they are reported as mean differences, slopes per year, etc., rather than using inferential statistics.”* In order to aid in the readability of descriptive summaries, I report the change of points per pages, per word counts, or over time.

**Comment 3:** If generalizing to "all newspapers," explicitly frame NYT as a sample and justify sampling assumptions.

**Response 3:** I now clarify that, while the New York Times represents a particular case study, it may also be considered as somewhat representative of the broader news environment given its influence. I add a new paragraph stating some of these considerations: *“It is a substantively important case study for understanding professional news language: it is one of the largest and most prominent news organizations in the United States, with a national and international readership and more than 12 million subscribers”* while qualifying that *“Although these features strengthen the usefulness of the case study, the New York Times is not necessarily representative of every newspaper or media outlet.”*

**Comment 4:** For survey data (which is a sample), retain inferential statistics but apply survey weights and report confidence intervals alongside p-values. The manuscript analyzes three nationally representative YouGov surveys without mentioning survey weights in Sections 3.4 or 4.3. Additionally, numerous statistical tests are conducted without correction for multiple comparisons. Apply appropriate survey weights to all YouGov regressions. Report full regression tables with standard errors, confidence intervals, and effect sizes (e.g., standardized coefficients or marginal effects). Apply a multiple-comparisons correction (e.g., False Discovery Rate, Bonferroni) given the number of tests across tables, OR explicitly state that findings are uncorrected exploratory results. Report model fit statistics such as R2 (squared R) and AIC/BIC, for all regression models

**Response 4:** In response to Comment 4 from Reviewer 1, I have removed the YouGov analyses from the paper, as you have each correctly noted that these analyses are underdeveloped and have a generally weak/tenuous conceptual connection to the *New York Times* analyses.

**Comment 5:** Section 3.1 describes concreteness scoring only at a high level, but the implementation details are missing, making the analysis non-reproducible. Specify tokenization and lemmatization rules (handling of punctuation, contractions, capitalization). Specify matching logic for overlapping unigrams and bigrams (priority, avoidance of double-counting) Treatment of out-of-vocabulary words (were they excluded or assigned neutral value?) Aggregation method such as mean, median, length-normalized. Coverage rates, i.e. percentage of tokens matched against dictionary, for each text type.

**Response 5:** I specify this now by stating that *“Before dictionary matching, all text was converted to lowercase and punctuation was removed, while contractions were retained because some contracted forms of words are included”* as well as that *“When a bigram appeared in the dictionary, the bigram score was prioritized and its constituent words were skipped as unigrams, preventing double counting.”* Other content was moved up from the Appendix and expanded, such as the elaboration that: *“Tokens not found in the dictionary were excluded from both the summed score and its denominator* *, to avoid influencing the mean response rating. Given the breadth of the dictionary, unmatched tokens consisted primarily of proper names, acronyms, and misspelled words, and pre-processing reduced mismatches caused by capitalization and punctuation.”* I also added the metric of coverage rate in the Methods section: “*Word coverage across the full New York Times database is fairly strong, with 73.8% of words having a direct match to the database (69% for headlines, 75% for abstract, 77% for lead paragraph).”*

**Comment 6:** Concreteness scores are bounded between 1 and 5. Linear regression assumes unbounded, normally distributed residual. Justify linear regression use for bounded dependent variables, OR consider beta regression / ordinal logistic alternatives. Provide diagnostic checks in appendix such as residual plots, Q-Q plots, homoscedasticity tests. Report whether assumptions are violated and how this affects interpretation.

**Response 6:** I appreciate the reviewer’s important methodological points. Because the statistics were replaced with population statistics (means/slopes), and the YouGov data were removed from analyses, linear regression is no longer used for hypothesis testing, so these checks were not necessary.

**Comment 7:** Although the submission form indicates data are fully available, the manuscript lacks a Data Availability Statement. Add a Data Availability Statement specifying the repository where computed concreteness scores, analysis code, and metadata will be deposited. Ensure all materials necessary for reproducibility are included, such as preprocessing scripts, scoring code, regression scripts and aggregated data files.

**Response 7:** The reviewer makes an excellent point. I have amended this by adding a Data Availability statement, along with a link where the materials necessary for reproducibility will be deposited.

**Comment 8:** Section 3.2 admits "No a priori hypotheses were formulated for these analyses," marking the work as exploratory. However, Discussion and Conclusions use definitive, causal language. Clearly label metadata analyses (Section 3.2) and demographic comparisons (Section 4.3) as exploratory. Temper causal language throughout (e.g., replace "drawing people away from" with "associated with" or "coincides with")

**Response 8:** The reviewer is correct that much of the language of the paper was too strong, given the exploratory nature of the analyses. I have made a concerted effort to temper causal language throughout the paper.

**Comment 9:** Add limitations paragraph acknowledging exploratory nature and need for pre-registered confirmatory studies

**Response 9:** I have now added a limitations paragraph explaining how the *New York Times* may not be representative and how alternative methodologies should be studied in future research.

**Comment 10:** Standardize figure numbering (Arabic vs. Roman numerals, e.g. Figure 2 vs. Figure II)

**Response 10:** Figure numbering was originally implemented as Arabic numerals for the main text and Roman numerals for the Appendix. However, I see how this format could be confusing. As such, I adjusted the numbering to be consistently Arabic numerals throughout.

**Comment 11:** Review ethical compliance for quoted survey responses (Section 4.2 contains potentially identifiable text with profanity)

**Response 11:** I appreciate the reviewer’s careful concern for potentially identifying material. This text has been removed as a consequence of narrowing the focus of the paper to the *New York Times*, but I will keep this in mind in my future research where I use individual level text data.

**Comment 12:** Proofread for grammatical errors ( "omissions within this database"), and consistency ("mass-level" vs. "mass level", "two-million", "front-page news" (hyphenated) vs. "front page news", etc). Awkward phrasing – Section 4.3: "Republicans in particular became significantly vivid with their language used to describe the "DEI" concept." Contradictory phrasing – Section 4.2: "The most concrete article headlines and individual-level responses were similar and different in several ways."

**Response 12:** I have now fixed these particular grammatical errors, as well as several others I noticed in the editing of the new manuscript.

**Comment 13:** The em‑dash should be paired with a second dash or replaced with commas. (Section 4.1: "Headlines – the shortest average...")

**Response 13:** I have now replaced this em dash with a comma.

**Additional Editor Comments**
**Comment 1:** Regarding the study by Crossley et al (2017) which you cite (page 3) about concreteness influencing text comprehension. In that particular study, concreteness was not indicated as such (it was not among the variables correlated with comprehension). There are other studies that show a relation of concreteness and text, but not that one.

**Response 1:** I appreciate the editor’s careful attention to the content of this citation. It has now been removed from the paper.

**Comment 2:** Regarding section 3.1 Computing Concreteness. I completely agree with the reviewer who asked for a detailed documentation of your NLP processing.

**Response 2:** In line with my response to those reviewer comments, I have added additional documentation and explanation of my NLP processing.

**Comment 3:** However, there might be a methodological problem with your approach. According to your illustration in figure 1, you seem to be utilizing concreteness ratings not only from content words, but also from function words, especially prepositions (like ‘of’) and determiners (like ‘the’). Since most of such words are quite abstract, including them in the counts skews the averages towards a more abstract side. You should seriously consider redoing your calculations while utilizing only content words, or at least using a stoplist of common function words.

**Response 3:** This is an excellent point. In order to test the robustness of these findings, I performed additional analyses on the average scores when only including content words, and when not including stop words. In both cases, none of the directions of results changed significantly. Accordingly, in the text I now state that: *“As an additional robustness test, the same analyses in the results section were also performed using a score that rated only content words and, separately, a score that did not rate stop words. In each case, none of the directions of the relationships changed, so the results going forward are reported from the ratings using the full corpus of words.”* I will include all of this code and alternative ratings in the replication materials so other scholars may also study any potential variations by rating style.

**Comment 4:** Page 7; “Headlines – the shortest average text snippets, tend to display more variance compared to abstract or lead paragraph text (Figure I).” Did you mean Figure 2?

**Response 4:** Originally, this was referring to Figure I, though now this is referring to the newly numbered Figure 4.
 **Comment 5:** Page 9: “Evidence suggests that print page has…” What is meant here by ‘print page’ ? Describe.

**Response 5:** To elaborate on this variable, I have now included clearer descriptions such as *“Page of the paper that the article is printed on,”* *“placement in the paper”* and *“page of the paper an article appears on.”*

**Comment 6:** Page 9: “For results of article word count…”. This is misleading. Since you did not actually analyze full articles, just some fields from scraped data, the notion ‘article word count’ is misleading here.

**Response 6:** I appreciate the editor’s attention to detail. In this database, article word count does actually refer to the full article’s word count as stored in metadata, even though the full text of the article is not available through the API. Because this is confusing, I have made this clearer in the text by adding a footnote stating that *“Though the full-article word count is present in metadata from the New York Times API, the full article text itself is not.”* and by differentiating *“word count”* of different metadata fields from *“full article word count.”*

**Comment 7:** Page 9: what does it mean “longer, later articles”? ‘longer’ suggests focus on article length, while ‘later’ suggests some time point. Since you are reporting t-tests, it seems you are comparing two groups in each comparison, like early and late articles? But relative to what point in time? You must describe in great detail what are the groups/samples/populations that you are comparing in each test.

**Response 7:** I clarified this language by describing in more elaborated terms: *“Together, these results suggest that longer articles and articles appearing later in the print edition may be more interpretive in their headlines and summaries…”* While I am no longer utilizing t-tests, I made efforts to describe the metadata fields in greater detail throughout. I have also attempted to better characterize comparisons between different categories of NYT metadata.

**Comment 8:** Page 9: “elite media headlines and public responses”. Di you mean NYT headlines vs. survey responses? Be more specific. Page 9: ‘The “woke” and “MAGA” concepts were most
frequently discussed in Opinion and Politics, while “DEI” also appeared prominently in Business Day and U.S. section’ This seems to suggest that you checked which articles (abstract/headlines) were discussing ‘woke’ or ‘MAGA’. How was this operationalized? How such topicness was defined? Did you search for the string ‘MAGA’? Can and abstract discuss DEI without explicitly using that acronym? You must describe your methodology in detail. Page 9: “The most concrete article headlines and individual-level responses were similar and different in several ways” How did you define “The most concrete…”? Most concrete in a list sorted by concreteness values? What size were those sets? What cutoff values were used for slicing?

**Response 8:** I appreciate the editor’s request for clarity in defining these concepts. Many of these issues were due to the relatively underdeveloped nature of the mass level section of the paper. These have been largely addressed by removing this content in response to Reviewer 1’s comments, though I have made efforts to improve methodological clarity in the *New York Times* analyses as well.

**Comment 9:** In general, your descriptions of data arrangements – like subgroup definitions and derivations, are inadequate and missing in detail, which also precludes from evaluating the adequacy of your methodology.

**Response 9:** I agree with the editor that there was a lack of precision in describing subgroups and methodologies regarding the prior survey data. Though I interpreted this comment as primarily concerned with the prior survey work, I have made efforts to more carefully define my descriptions of variables in the new draft.

**Comment 10:** Section 4.3 is peppered with seemingly random comparisons. For example, Democrats are compared to Republicans (what numbers is not reported) on specific dates. But no background is given why those particular dates are chosen for reporting. Regarding the response ‘I don’t know’ – instead of dwelling on this as ‘the most abstract’ response, you should probably exclude such responses from your data, as they reflect a technical avoidance of the topic. Regarding predictors of concreteness in survey data – where did the predictor variables come from? From you-gov? Self-reported? Also provide descriptive statistics about respondents and their variables.

**Response 10:** I agree with the Editor that the comparisons across datasets were not well aligned and were described in confusing ways. I also agree with the assessment that a response like “I don’t know” might not necessarily constitute real abstraction. In the NYTimes data, these are fewer common issues, but I believe the Editor’s comment to analyze by not counting stop words and/or counting content words allowed me to better make measured claims relating to these more vague operationalizations of abstraction.

**Comment 11:** Precision of descriptions. Please be much more precise with the descriptions used in the manuscript. For example, page 14: “Terms like “woke” were rated as significantly more concrete among non-white respondents”. You did not conduct a rating study on the concreteness of the term ‘woke’ (or other terms). You were measuring the average concreteness of verbal responses for descriptive survey questions. You might say “ ‘woke’ was described on average in much more concrete terms…” You should not say ‘rated’. This kind of sloppiness occurs in other parts of the manuscript as well.

**Response 11:** The editor is correct in this assessment. While many of these errors were concentrated in the survey and survey/elite comparison text, I found many of these inconsistencies in the broader text and have made efforts to reduce them.
