# Supplementary material for: The weight of words: Applying concreteness to elite media language
Source: PLoS One. 2026 Oct 1;21(10):e0359092. doi: 10.1371/journal.pone.0359092 (PMC13630233; doi:10.1371/journal.pone.0359092)
Supplement: Supplementary file 3 [file pone.0359092.s003.docx]

Response to Reviewers, Editor

I thank the editor and the reviewers for their thoughtful comments throughout this process of revision. My responses to the remaining comments are below:

**Reviewer Comments**

**Comment 1:** Minor issues remain. The author replaced inferential statistics with descriptive summaries for the NYT population data, which was the central statistical concern raised in the previous round. The population-framing rationale is well stated, the NLP pipeline is now reproducible, causal language has been meaningfully tempered throughout, and figure numbering is consistent. The survey analyses and t-tests also appear to have been cleanly removed.
Three minor issues remain. First, the abstract still uses “demonstrate” where the body now correctly uses “suggest.”

**Response 1:** I appreciate the reviewer’s careful attention to detail. I have now corrected this word.

**Comment 2:** The Introduction retains a reference to "open-ended survey responses" that conflicts with the revised NYT-only scope.

**Response 2:** I examined the paper and was unable to find this reference – is it possible that this was a relic from the “track changes” version of the manuscript?

**Comment 3:** Second, the Data Availability Statement should enumerate specific deposited materials (preprocessing scripts, scoring code, aggregated score files) and confirm the repository is already populated.

**Response 3:** I have updated this language to be more specific, now statin*g: “The data and replication materials used in this study are available at https://osf.io/w3eqr/. This contains the Python code used to score concreteness, versions of the New York Times data that were scraped using the API, and R code that was used to perform the analyses in the paper.”*

**Comment 4:** Third, the limitations paragraph should include one sentence recommending pre-registered designs for future confirmatory research, as originally requested.

**Response 4:** I appreciate the reviewer’s suggestion, I have now expanded a sentence in the limitations paragraph, that now says: *“Analyses of other news sources and alternative methodologies could better illuminate the concreteness construct in the broader media environment, and could provide pre-registered, confirmatory research that would supplement the descriptive analyses of this paper.”*
