# Supplementary material for: The weight of words: Applying concreteness to elite media language
Source: PLoS One. 2026 Oct 1;21(10):e0359092. doi: 10.1371/journal.pone.0359092 (PMC13630233; doi:10.1371/journal.pone.0359092)
Supplement: Peer review history

# Peer review: Decision Letter 0

**DOI:** 10.1371/journal.pone.0359092.r001

**Authors:** Michael Flor

7 Apr 2026

Dear Dr. Snyder,

Thank you for submitting your manuscript to PLOS ONE. After careful consideration, we feel that it has merit but does not fully meet PLOS ONE’s publication criteria as it currently stands. Therefore, we invite you to submit a revised version of the manuscript that addresses the points raised during the review process.

Please submit your revised manuscript by May 21 2026 11:59PM. If you will need more time than this to complete your revisions, please reply to this message or contact the journal office at plosone@plos.org. When you're ready to submit your revision, log on to https://www.editorialmanager.com/pone/ and select the 'Submissions Needing Revision' folder to locate your manuscript file.

- A letter that responds to each point raised by the academic editor and reviewer(s). You should upload this letter as a separate file labeled 'Response to Reviewers'.
- A marked-up copy of your manuscript that highlights changes made to the original version. You should upload this as a separate file labeled 'Revised Manuscript with Track Changes'.
- An unmarked version of your revised paper without tracked changes. You should upload this as a separate file labeled 'Manuscript'.

If you would like to make changes to your financial disclosure, please include your updated statement in your cover letter. Guidelines for resubmitting your figure files are available below the reviewer comments at the end of this letter.

If applicable, we recommend that you deposit your laboratory protocols in protocols.io to enhance the reproducibility of your results. Protocols.io assigns your protocol its own identifier (DOI) so that it can be cited independently in the future. For instructions see: https://journals.plos.org/plosone/s/submission-guidelines#loc-laboratory-protocols. Additionally, PLOS ONE offers an option for publishing peer-reviewed Lab Protocol articles, which describe protocols hosted on protocols.io. Read more information on sharing protocols at https://plos.org/protocols?utm_medium=editorial-email&utm_source=authorletters&utm_campaign=protocols.

As the corresponding author, your ORCID iD is verified in the submission system and will appear in the published article. PLOS supports the use of ORCID, and we encourage all coauthors to register for an ORCID iD and use it as well. Please encourage your coauthors to verify their ORCID iD within the submission system before final acceptance, as unverified ORCID iDs will not appear in the published article. *Only*  the individual author can complete the verification step; PLOS staff *cannot*  verify ORCID iDs on behalf of authors.

We look forward to receiving your revised manuscript.

Kind regards,

Michael Flor

Academic Editor

PLOS One

Journal requirements:

When submitting your revision, we need you to address these additional requirements.

1. Please ensure that your manuscript meets PLOS ONE's style requirements, including those for file naming. The PLOS ONE style templates can be found at

https://journals.plos.org/plosone/s/file?id=wjVg/PLOSOne_formatting_sample_main_body.pdf and

https://journals.plos.org/plosone/s/file?id=ba62/PLOSOne_formatting_sample_title_authors_affiliations.pdf

2. When completing the data availability statement of the submission form, you indicated that you will make your data available on acceptance. We strongly recommend all authors decide on a data sharing plan before acceptance, as the process can be lengthy and hold up publication timelines. Please note that, though access restrictions are acceptable now, your entire data will need to be made freely accessible if your manuscript is accepted for publication. This policy applies to all data except where public deposition would breach compliance with the protocol approved by your research ethics board. If you are unable to adhere to our open data policy, please kindly revise your statement to explain your reasoning and we will seek the editor's input on an exemption. Please be assured that, once you have provided your new statement, the assessment of your exemption will not hold up the peer review process.

3. Please update your submission to use the PLOS LaTeX template. The template and more information on our requirements for LaTeX submissions can be found at http://journals.plos.org/plosone/s/latex.

4. Please include your full ethics statement in the ‘Methods’ section of your manuscript file. In your statement, please include the full name of the IRB or ethics committee who approved or waived your study, as well as whether or not you obtained informed written or verbal consent. If consent was waived for your study, please include this information in your statement as well.

5. Please include captions for your Supporting Information files at the end of your manuscript, and update any in-text citations to match accordingly. Please see our Supporting Information guidelines for more information: http://journals.plos.org/plosone/s/supporting-information.

If the reviewer comments include a recommendation to cite specific previously published works, please review and evaluate these publications to determine whether they are relevant and should be cited. There is no requirement to cite these works unless the editor has indicated otherwise.

**Additional Editor Comments**

===========================================

Please pay close attention to reviewers comments.

Here are some additional comments from the editor:

[1] (minor comment)

Regarding the study by Crossley et al (2017) which you cite (page 3) about concreteness influencing text comprehension. In that particular study, concreteness was not indicated as such (it was not among the variables correlated with comprehension). There are other studies that show a relation of concreteness and text, but not that one.

[2] (major comment)

Regarding section 3.1 Computing Concreteness.

I completely agree with the reviewer who asked for a detailed documentation of your NLP processing.

However, there might be a methodological problem with your approach. According to your illustration in figure 1, you seem to be utilizing concreteness ratings not only from content words, but but also from function words, especially prepositions (like ‘of’) and determiners (like ‘the’). Since most of such words are quite abstract, including them in the counts skews the averages towards a more abstract side. You should seriously consider redoing you calculations while utilizing only content words, or at least using a stoplist of common function words.

[3] (minor comment)

Page 7; “Headlines – the shortest average text snippets,

tend to display more variance compared to abstract or lead paragraph text (Figure I).”

Did you mean Figure 2?

[4] (minor comment)

Page 9: “Evidence suggests that print page has…” What is meant here by ‘print page’ ? Describe.

[5]

Page 9: “For results of article word count…”. This is misleading. Since you did not actually analyze full articles, just some fields from scraped data, the notion ‘article word count’ is misleading here.

[6]

Page 9:

what does it mean “longer, later articles”? ‘longer’ suggests focus on article length, while ‘later’ suggests some time point. Since you are reporting t-tests, it seems you are comparing two groups in each comparison, like early and late articles? But relative to what point in time? You must describe in great detail what are the groups/samples/populations that you are comparing in each test.

[7]

Page 9: “elite media headlines and public responses”. Di you mean NYT headlines vs. survey responses? Be more specific.

[8]

Page 9: ‘The “woke” and “MAGA” concepts were most

frequently discussed in Opinion and Politics, while “DEI” also appeared prominently in

Business Day and U.S. section’

This seems to suggest that you checked which articles (abstract/headlines) were discussing ‘woke’ or ‘MAGA’. How was this operationalized? How such topicness was defined? Did you search for the string ‘MAGA’? Can and abstract discuss DEI without explicitly using that acronym? You must describe your methodology in detail.

[9]

Page 9:

“The most concrete article headlines and individual-level responses were similar and different in several ways”

How did you define “The most concrete…”? Most concrete in a list sorted by concreteness values? What size were those sets? What cutoff values were used for slicing?

Major point:

In general, your descriptions of data arrangements – like subgroup definitions and derivations, are inadequate and missing in detail, which also precludes from evaluating the adequacy of your methodology.

Section 4.3 is peppered with seemingly random comparisons. For example, Democrats are compared to Republicans (what numbers is not reported) on specific dates. But no background is given why those particular dates are chosen for reporting.

[10]

Regarding the response ‘I don’t know’ – instead of dwelling on this as ‘the most abstract’ response, you should probably exclude such responses from you data, as they reflect a technical avoidance of the topic.

[11]

Regarding predictors of concreteness in survey data – where did the predictor variables come from? From you-gov? Self-reported? Also provide descriptive statistics about respondents and their variables.

[12]

Precision of descriptions. Please be much more precise with the descriptions used in the manuscript.

For example, page 14: “Terms like “woke” were rated as significantly more concrete among non-white respondents”. You did not conduct a rating study on the concreteness of the term ‘woke’ (or other terms). You were measuring the average concreteness of verbal responses for descriptive survey questions. You might say “ ‘woke’ was described on average in much more concrete terms…” You should not say ‘rated’. This kind of sloppiness occurs in other parts of the manuscript as well.

===========================================

[Note: HTML markup is below. Please do not edit.]

Reviewers' comments:

Reviewer's Responses to Questions

**Comments to the Author**

1. Is the manuscript technically sound, and do the data support the conclusions?

Reviewer #1: Partly

Reviewer #2: Partly

**********

2. Has the statistical analysis been performed appropriately and rigorously?

Reviewer #1: Yes

Reviewer #2: No

**********

3. Have the authors made all data underlying the findings in their manuscript fully available?

The PLOS Data policy

Reviewer #1: Yes

Reviewer #2: No

**********

4. Is the manuscript presented in an intelligible fashion and written in standard English?

Reviewer #1: Yes

Reviewer #2: Yes

**********

Reviewer #1: This is a very interesting article, of relevance to public opinion research, as well as to political science, discourse and communication. The data and analysis concerning NYT articles seems to me to be very clear, except that I do not understand what you mean by "abstract" (is this an American term?). What is the "abstract" of a news or opinion article? This can easily be explained.

However, I have a few bigger concerns:

1. The NYT study is very interesting. But this goes from 1999 to 2026. Can you say more about the sudden change that seems to occur around 2005? Why don't you show the data for news and opinion articles separately? Surely the results must be very different.

2. Since the data from NYT cover 27 years, why is it that the opinion poll data only deal with 2024 and 2025? I do not understand the study design here, and it is not properly explained. This "opinion" study seems quite separate from the other one - unless, of course, you are simply testing the construct, but I think that is not the case.

3. Your discussion talks about the drop in concreteness in relation to " temporal changes in the media landscape". There is a vast literature on this (trivialisation, tabloidisation, effect of social media) that you need to address.

Personally, I feel that you could expand the discussion of the NYT data and leave the opinion poll data for another paper.

Reviewer #2: The manuscript introduces a valuable application of psycholinguistic concreteness to political text analysis. The core research question is whether concreteness captures meaningful stylistic differences in elite and mass-level political

language, wich is potentially significant for the field. The data sources (New York Times corpus, YouGov surveys) are well-established.

The manuscript has strong potential to establish concreteness as a standard tool in political text analysis. The core contribution is valid and valuable, and all remaining issues should be correctable through re-analysis, additional documentation, and tempered conclusions.

Methodological and statistical issues must be addressed before publication to meet the standards for rigor, transparency, and reproducibility, and below are specific required revisions:

1. STATISTICAL REPORTING FOR POPULATION DATA

The New York Times corpus (~2 million articles from 2000–2025) represents a population, not a sample, for claims specifically about NYT articles. Inferential statistics (p-values, t-tests) are conceptually inappropriate for population data because there is no sampling uncertainty to estimate.

- Remove t-tests and p-values for all NYT population-level analyses (Section 4.1)

- Replace with descriptive summaries: mean differences, slopes per year, and confidence intervals for measurement precision (not hypothesis testing)

- If generalizing to "all newspapers," explicitly frame NYT as a sample and justify sampling assumptions

- For survey data (which is a sample), retain inferential statistics but apply survey weights and report confidence intervals alongside p-values

2. SURVEY ANALYSIS STANDARDS

The manuscript analyzes three nationally representative YouGov surveys without mentioning survey weights in Sections 3.4 or 4.3. Additionally, numerous statistical tests are conducted without correction for multiple comparisons.

- Apply appropriate survey weights to all YouGov regressions

- Report full regression tables with standard errors, confidence intervals, and effect sizes (e.g., standardized coefficients or marginal effects)

- Apply a multiple-comparisons correction (e.g., False Discovery Rate, Bonferroni) given the number of tests across tables, OR explicitly state that findings are uncorrected exploratory results

- Report model fit statistics such as R2 (squared R) and AIC/BIC, for all regression models

3. NLP METHODOLOGY DOCUMENTATION

Section 3.1 describes concreteness scoring only at a high level, but the implementation details are missing, making the analysis non-reproducible.

- Specify tokenization and lemmatization rules (handling of punctuation, contractions,

capitalization)

- Specify matching logic for overlapping unigrams and bigrams (priority, avoidance of double-counting)

- Treatment of out-of-vocabulary words (were they excluded or assigned neutral value?)

- Aggregation method such as mean, median, length-normalized

- Coverage rates, i.e. percentage of tokens matched against dictionary, for each text type

4. MODEL SPECIFICATION

Concreteness scores are bounded between 1 and 5. Linear regression assumes unbounded, normally distributed residuals.

- Justify linear regression use for bounded dependent variables, OR consider beta regression / ordinal logistic alternatives

- Provide diagnostic checks in appendix such as residual plots, Q-Q plots, homoscedasticity tests

- Report whether assumptions are violated and how this affects interpretation

5. DATA AVAILABILITY

Although the submission form indicates data are fully available, the manuscript lacks a Data Availability Statement.

- Add a Data Availability Statement specifying the repository where computed concreteness scores, analysis code, and metadata will be deposited

- Ensure all materials necessary for reproducibility are included, such as preprocessing scripts, scoring code, regression scripts and aggregated data files.

6. INTERPRETATION AND LANGUAGE

Section 3.2 admits "No a priori hypotheses were formulated for these analyses," marking the work as exploratory. However, Discussion and Conclusions use definitive, causal language.

- Clearly label metadata analyses (Section 3.2) and demographic comparisons (Section 4.3) as exploratory

- Temper causal language throughout (e.g., replace "drawing people away from" with "associated with" or "coincides with")

- Add limitations paragraph acknowledging exploratory nature and need for

pre-registered confirmatory studies

MINOR REVISIONS:

1. Standardize figure numbering (Arabic vs. Roman numerals, e.g. Figure 2 vs. Figure II)

2. Review ethical compliance for quoted survey responses (Section 4.2 contains potentially identifiable text with profanity)

3. Proofread for grammatical errors ( "omissions within this database"), and consistency ("mass-level" vs. "mass level", "two-million", "front-page news" (hyphenated) vs. "front page news", etc)

4. Awkward phrasing – Section 4.3: "Republicans in particular became significantly vivid with their language used to describe the "DEI" concept."

5. Contradictory phrasing – Section 4.2: "The most concrete article headlines and individual-level responses were similar and different in several ways."

6. The em‑dash should be paired with a second dash or replaced with commas. (Section 4.1: "Headlines – the shortest average...")

**********

what does this mean?). If published, this will include your full peer review and any attached files.

If you choose “no”, your identity will remain anonymous but your review may still be made public.

**Do you want your identity to be public for this peer review?**  For information about this choice, including consent withdrawal, please see our Privacy Policy

Reviewer #1: No

Reviewer #2: No

**********

[NOTE: If reviewer comments were submitted as an attachment file, they will be attached to this email and accessible via the submission site. Please log into your account, locate the manuscript record, and check for the action link "View Attachments". If this link does not appear, there are no attachment files.]

To ensure your figures meet our technical requirements, please review our figure guidelines: https://journals.plos.org/plosone/s/figures

You may also use PLOS’s free figure tool, NAAS, to help you prepare publication quality figures: https://journals.plos.org/plosone/s/figures#loc-tools-for-figure-preparation.

NAAS will assess whether your figures meet our technical requirements by comparing each figure against our figure specifications.

# Author comment: Author response to Decision Letter 1

**DOI:** 10.1371/journal.pone.0359092.r002

23 Jul 2026

I have attached a response document in my files.

Attachment Submitted filename: Response to Reviewers, Editor.docx

# Peer review: Decision Letter 1

**DOI:** 10.1371/journal.pone.0359092.r003

**Authors:** Michael Flor

20 Aug 2026

Dear Dr. Snyder,

Please submit your revised manuscript by Oct 04 2026 11:59PM. If you will need more time than this to complete your revisions, please reply to this message or contact the journal office at plosone@plos.org. When you're ready to submit your revision, log on to https://www.editorialmanager.com/pone/ and select the 'Submissions Needing Revision' folder to locate your manuscript file.

- A letter that responds to each point raised by the academic editor and reviewer(s). You should upload this letter as a separate file labeled 'Response to Reviewers'.
- A marked-up copy of your manuscript that highlights changes made to the original version. You should upload this as a separate file labeled 'Revised Manuscript with Track Changes'.
- An unmarked version of your revised paper without tracked changes. You should upload this as a separate file labeled 'Manuscript'.

If you would like to make changes to your financial disclosure, please include your updated statement in your cover letter. Guidelines for resubmitting your figure files are available below the reviewer comments at the end of this letter.

If applicable, we recommend that you deposit your laboratory protocols in protocols.io to enhance the reproducibility of your results. Protocols.io assigns your protocol its own identifier (DOI) so that it can be cited independently in the future. For instructions see: https://journals.plos.org/plosone/s/submission-guidelines#loc-laboratory-protocols. Additionally, PLOS One offers an option for publishing peer-reviewed Lab Protocol articles, which describe protocols hosted on protocols.io. Read more information on sharing protocols at https://plos.org/protocols?utm_medium=editorial-email&utm_source=authorletters&utm_campaign=protocols.

As the corresponding author, your ORCID iD is verified in the submission system and will appear in the published article. PLOS supports the use of ORCID, and we encourage all coauthors to register for an ORCID iD and use it as well. Please encourage your coauthors to verify their ORCID iD within the submission system before final acceptance, as unverified ORCID iDs will not appear in the published article. *Only*  the individual author can complete the verification step; PLOS staff *cannot*  verify ORCID iDs on behalf of authors.

We look forward to receiving your revised manuscript.

Kind regards,

Michael Flor

Academic Editor

PLOS One

Journal Requirements:

If the reviewer comments include a recommendation to cite specific previously published works, please review and evaluate these publications to determine whether they are relevant and should be cited. There is no requirement to cite these works unless the editor has indicated otherwise.

Please review your reference list to ensure that it is complete and correct. If you have cited papers that have been retracted, please include the rationale for doing so in the manuscript text, or remove these references and replace them with relevant current references. Any changes to the reference list should be mentioned in the rebuttal letter that accompanies your revised manuscript. If you need to cite a retracted article, indicate the article’s retracted status in the References list and also include a citation and full reference for the retraction notice.

Additional Editor Comments :

The revised version of the manuscript is much improved.

I agree that the survey data was not yet 'well researched', and so dropping that aspect contributes to making the manuscript much more focused.

Overall, the manuscript is good now.

One of the reviewers has three comments, and I provide my comments on those.

1a.

'the abstract still uses "demonstrate" where the body now correctly uses "suggest" '

I agree - please change "demonstrate" to "suggest'.

1.b

'the Introduction retains a reference to "open-ended survey responses"... '

I checked the new introduction and did not find the phrase there. maybe the reviewer looked in the tracked-changes version.

Anyway, check it.

2.

The Data Availability Statement.

You provided a link to an OSF repository.

I logged into OSF, but could not access the repository, OSF claims I don't have permission.

Please check that aspect.

3.

"the limitations paragraph should include one sentence recommending preregistered

designs for future confirmatory research".

I am not a big fan of pre-registration, but as the reviewer asked for this minor change, please consider to add it.

[Note: HTML markup is below. Please do not edit.]

Reviewers' comments:

Reviewer's Responses to Questions

**Comments to the Author**

Reviewer #1: All comments have been addressed

Reviewer #2: (No Response)

**********

2. Is the manuscript technically sound, and do the data support the conclusions?

Reviewer #1: (No Response)

Reviewer #2: Partly

**********

3. Has the statistical analysis been performed appropriately and rigorously?

Reviewer #1: (No Response)

Reviewer #2: Yes

**********

4. Have the authors made all data underlying the findings in their manuscript fully available?

The PLOS Data policy

Reviewer #1: (No Response)

Reviewer #2: Yes

**********

5. Is the manuscript presented in an intelligible fashion and written in standard English?

Reviewer #1: (No Response)

Reviewer #2: Yes

**********

Reviewer #1: (No Response)

Reviewer #2: Minor issues remain. The author replaced inferential statistics with descriptive summaries for the NYT population data, which was the central statistical concern raised in the previous round. The population-framing rationale is well stated, the NLP pipeline is now reproducible, causal language has been meaningfully tempered throughout, and figure numbering is consistent. The survey analyses and t-tests also appear to have been cleanly removed.

Three minor issues remain. First, the abstract still uses "demonstrate" where the body now correctly uses "suggest," and the Introduction retains a reference to "open-ended survey responses" that conflicts with the revised NYT-only scope. Second, the Data Availability Statement should enumerate specific deposited materials (preprocessing scripts, scoring code, aggregated score files) and confirm the repository is already populated. Third, the limitations paragraph should include one sentence recommending pre-registered designs for future confirmatory research, as originally requested.

**********

what does this mean?). If published, this will include your full peer review and any attached files.

If you choose “no”, your identity will remain anonymous but your review may still be made public.

**Do you want your identity to be public for this peer review?**  For information about this choice, including consent withdrawal, please see our Privacy Policy

Reviewer #1: No

Reviewer #2: No

**********

[NOTE: If reviewer comments were submitted as an attachment file, they will be attached to this email and accessible via the submission site. Please log into your account, locate the manuscript record, and check for the action link "View Attachments". If this link does not appear, there are no attachment files.]

To ensure your figures meet our technical requirements, please review our figure guidelines: https://journals.plos.org/plosone/s/figures

You may also use PLOS’s free figure tool, NAAS, to help you prepare publication quality figures: https://journals.plos.org/plosone/s/figures#loc-tools-for-figure-preparation.

NAAS will assess whether your figures meet our technical requirements by comparing each figure against our figure specifications.

# Author comment: Author response to Decision Letter 2

**DOI:** 10.1371/journal.pone.0359092.r004

31 Aug 2026

I have added my response to reviewers as an attached file.

Attachment Submitted filename: Response to Reviewers.docx

# Editor report: Decision Letter 2

**DOI:** 10.1371/journal.pone.0359092.r005

**Authors:** Michael Flor

10 Sep 2026

The Weight of Words: Applying Concreteness to Elite Media Language

PONE-D-26-08711R2

Dear Dr. Snyder,

We’re pleased to inform you that your manuscript has been judged scientifically suitable for publication and will be formally accepted for publication once it meets all outstanding technical requirements.

Within one week, you’ll receive an e-mail detailing the required amendments. When these have been addressed, you’ll receive a formal acceptance letter and your manuscript will be scheduled for publication.

An invoice will be generated when your article is formally accepted. Please note, if your institution has a publishing partnership with PLOS and your article meets the relevant criteria, all or part of your publication costs will be covered. Please make sure your user information is up-to-date by logging into Editorial Manager at Editorial Manager® and clicking the ‘Update My Information' link at the top of the page. For questions related to billing, please contact billing support.

If your institution or institutions have a press office, please notify them about your upcoming paper to help maximize its impact. If they’ll be preparing press materials, please inform our press team as soon as possible -- no later than 48 hours after receiving the formal acceptance. Your manuscript will remain under strict press embargo until 2 pm Eastern Time on the date of publication. For more information, please contact onepress@plos.org.

Kind regards,

Michael Flor

Academic Editor

PLOS One

Additional Editor Comments (optional):

Reviewers' comments:

# Editor report: Acceptance letter

**DOI:** 10.1371/journal.pone.0359092.r006

**Authors:** Michael Flor

PONE-D-26-08711R2

PLOS One

Dear Dr. Snyder,

I'm pleased to inform you that your manuscript has been deemed suitable for publication in PLOS One. Congratulations! Your manuscript is now being handed over to our production team.

At this stage, our production department will prepare your paper for publication. This includes ensuring the following:

* All references, tables, and figures are properly cited

* All relevant supporting information is included in the manuscript submission,

* There are no issues that prevent the paper from being properly typeset

You will receive further instructions from the production team, including instructions on how to review your proof when it is ready. Please keep in mind that we are working through a large volume of accepted articles, so please give us a few days to review your paper and let you know the next and final steps.

Lastly, if your institution or institutions have a press office, please let them know about your upcoming paper now to help maximize its impact. If they'll be preparing press materials, please inform our press team within the next 48 hours. Your manuscript will remain under strict press embargo until 2 pm Eastern Time on the date of publication. For more information, please contact onepress@plos.org.

You will receive an invoice from PLOS for your publication fee after your manuscript has reached the completed accept phase. If you receive an email requesting payment before acceptance or for any other service, this may be a phishing scheme. Learn how to identify phishing emails and protect your accounts at https://explore.plos.org/phishing.

If we can help with anything else, please email us at customercare@plos.org.

Thank you for submitting your work to PLOS One and supporting open access.

Kind regards,

PLOS One Editorial Office Staff

on behalf of

Dr. Michael Flor

Academic Editor

PLOS One
